# Supplementary material for: Are carbon emissions trading and green financial instruments synergistic? -Comprehensive quantitative research based on content analysis
Source: PLoS One. 2024 Mar 7;19(3):e0298601. doi: 10.1371/journal.pone.0298601 (PMC10919601; doi:10.1371/journal.pone.0298601)
Supplement: S1 Data — (ZIP) [file pone.0298601.s001.zip › Data/地级市Z_F工作报告.zip!/╣π╢1⁄2/╓╨╔╜╩╨/└┤╫╘╣1⁄2╓┌║┼í╛┬φ┐╦╩2╛▌═°í┐_2005.docx]

各位代表：

我代表市人民政府向大会作政府工作报告，请予审议，并请政协委员和其他列席人员提出意见。

2004年工作回顾

2004年是本届政府的开局之年。在省委、省政府和市委的正确领导下，在市人大及其常委会和市政协的监督支持下，市政府坚持以邓小平理论和“三个代表”重要思想为指导，深入贯彻十六大和十六届三中、四中全会、省委九届四次全会和市委十一届一次全会精神，全面实施经济社会协调发展战略，贯彻中央宏观调控政策，开拓进取，迎难而上，克服了能源紧缺和原材料价格上涨等因素的影响，经济持续快速健康发展，各项社会事业全面进步，人民生活水平稳步提高。圆满完成了十二届人大一次会议提出的各项经济社会预期目标。全市生产总值610.1亿元，增长18.7%；财政一般预算收入34.5亿元（新口径），增长19.9%；全社会固定资产投资291.3亿元，增长11.2%；社会消费品零售总额178.8亿元，增长16.4%；年末各项存款余额达959.7亿元，比年初增长13.5%。

产业实力明显增强，经济质量再上台阶。坚持“工业强市”发展战略，工业发展保障体系进一步完善，发展后劲增强，实现工业总产值2004.4亿元，增长27.8%。消化盘活各类闲置土地1.2万亩，园区集约化、专业化水平进一步提高。结构调整步伐加快，高新技术产品产值占全市工业总产值的22.3%，规模以上五大支柱产业占工业总产值56.4%，装备制造业产值达404.1亿元，国家级专业产业基地增至18个。名牌带动战略取得新成效，新增中国名牌产品2个，中国驰名商标2个，国家免检产品6个，广东省著名商标及名牌产品共47个。依靠先进技术改造传统产业取得实效，企业技改项目232个，总投资52.9亿元。第三产业特别是连锁、仓储式销售等现代流通业稳步发展，加快建设以区域经济为依托的物流市场，一批大型流通企业快速发展壮大。加大旅游促销工作力度，完善旅游“六要素”，旅游业总收入57.2亿元，增长16.4%。房地产业和金融、证券、保险业健康发展，各类中介服务进一步规范。商品流通和社会消费活跃，居民消费价格总指数103.1%。

农业农村经济加快发展，农民收入稳步提高。实现农业总产值49.5亿元，增长5.4%；农村经济总收入1336.7亿元，增长21.9%。进一步调整农业产业结构，高产、优质、高效农业加快发展，农业龙头企业14家，实现销售收入9.8亿元。恢复和发展粮食生产，全年粮食播种面积39.6万亩，总产量14.5万吨，全面完成省下达的各项粮食指标。积极培育农产品知名品牌，5个农产品通过国家A级绿色食品认证，新增广东省（农业）名牌产品8个。强化农业基础设施建设，农村“五项工程”共投入1.99亿元，新建4米宽以上农路262.9公里，农桥（涵）177座，整治低产鱼塘4万亩，整治内河175公里，“一区三线”森林改造工程第五期植树1万多亩，长江库区生态林改造第一期4833亩，“四旁”绿化种植长度296公里。积极深化农村各项改革，114个村（居）完成农村股份合作制改革，实施农村税费改革，减轻农民负担1.24亿元。加强农村基层建设，进一步规范村级干部工作报酬，农村大局稳定。

基础设施和重点项目全面推进。完成全市城市空间发展概念规划，启动新一轮城市总体规划修编。按可持续发展的要求，组织编制人口、水资源、环境保护、交通和组团发展等五个专项规划。中心镇加快发展，四大组团发展框架基本形成，城市化水平达60.7%。按照“内通外拓”的思路，进一步完善交通基础设施，南北外环路、古神公路、城桂公路二期建设工程全面启动。实施公交优先，公交事业有新发展。城市基础设施建设加快，完成中心组团垃圾综合处理基地垃圾填埋场和污水处理厂工程、长江水厂等重点建设工程，中嘉污水处理厂二期工程、珍家山污水处理厂首期、市疾病预防控制中心、市第二人民医院、中心粮库等项目正加紧施工。完成香山500千伏、同益、旗乐、逸仙220千伏和永宁、文安、泮沙110千伏输变电工程，南朗电厂建成投运，永安电厂基本完工。信息化水平进一步提高，智能公交第一期工程、中山市行政服务在线系统建成投入使用。经营城市取得新成效，土地储备力度加大。积极做好历史用地善后和问题楼盘处置工作。组建城市建设投资集团有限公司，多渠道筹集建设资金。综合执法体系逐步完善，建立综合协调与行政执法并举的城管工作体制，执法重心下移，城市环境质量和管理水平进一步提高。

内外源经济协调发展。民营经济发展迅猛，活力增强。新增民营企业2200多家，个体工商户9300多户。民营工业企业实现产值743.1亿元，民营经济对全市经济贡献率达45.5%。民营企业上市融资取得突破，华帝股份有限公司成功上市。外贸出口保持较快增长，实现外贸出口总值99.9亿美元，增长21.1%。出口商品的质量档次有所提高。加强口岸配套设施建设，口岸现代化管理和大通关建设取得新成绩。注重引进外资质量，全年实际利用外资5.25亿美元，增长43%。新增直接外商投资企业355家。成功举办3·28招商经贸洽谈会、轻博会、服装节、灯博会、电展会等系列活动。

各项改革取得新进展。初步建立起公共财政基本框架。加大公有产权制度以及公有企业改革力度，成立国资委，依法履行公有资产监督营运、监督管理职责，建立考核奖励制度，列入考核类公有企业利润增长28.6%。大力盘活公有资产。拓宽财政增收渠道，非税收入30.1亿元。加强支出管理，开展财政支出绩效评价。进一步理顺市镇财税管理体制，安排财政转移支付资金5000万元。完成市直机关首批公车改革。大力整顿和规范市场经济秩序，重点实施食品安全工程，专项整治成效显著，市场环境明显改善。全面推进社会信用体系建设，制定公务员公约、青年公约、妇女公约，中山信用网如期开通。深化中小学人事制度改革，完成校长职级制改革和首届中山名校长、名教师评选工作。

各项社会事业全面进步。大力推进教育强市战略，9个镇区通过了省的教育强镇（区）验收，教育强镇总数达12个。启动振兴初中行动计划。教育质量和办学水平全面提高，重点本科录取1023人。高等教育进一步发展。教育布局调整工作全面开展，各级投入资金4.3亿元，对105所学校进行了布局调整，上等级学校达160所。共投入6100万元加速教育信息化建设，114所学校实现“校校通”。民办教育取得新发展，民办中小学共有45所。科技事业进一步发展。企业科技创新能力增强，有46家企业通过省级高新技术企业考核，3家荣获“省级优秀高新技术企业”称号，省级高新技术企业共104家。获得国家、省科技项目44项，4项成果获得省科学技术奖。市级科技三项经费1561万元，技改贴息3910万元。推动民营经济科技进步和创新，12家民科企业获“中国优秀民营科技企业”称号，民科园被批准为“国家级民营科技园”。继续完善人才“柔性流动”政策，新引进各类人才13万人（新口径）。文化事业进一步发展，加快建设文化艺术中心、商业博物馆，开展名人故居及文物修缮工作，广播电视、新闻出版、图书音像市场健康发展，市图书馆顺利通过国家一级馆的评估验收。“好山好水好儿女”中山籍文化名人省亲活动取得圆满成功。加强社区体育设施建设，兴建全民健身广场二期工程，全民健身运动蓬勃开展。火炬开发区荣获“全国亿万农民健身活动先进乡镇”和“全国城市体育先进社区”称号。竞技体育水平不断提高，获得全国冠军24个，成功承办了广东省第八届中学生运动会。加强公共卫生体系建设，防控重大卫生事件能力增强。医疗卫生质量有新的提高。大力开展创建卫生镇村工作，现有国家卫生镇6个、省卫生镇13个。新型农村合作医疗工作有新发展，参加人口占农业总人口的94.3%，社区卫生服务不断拓展。全市常住人口出生率为10.48‰，计划生育率为93.95%。加强民兵预备役建设和国防教育，双拥工作再创佳绩，荣获“全国双拥模范城”称号。启动争创全国文明城市工作。大力开展社会治安综合治理，“严打”取得新成效。落实各级信访责任，切实抓好安全生产。

人民生活水平和生活质量不断提高。城镇居民年人均可支配收入15836元，增长6.2%；农民人均年纯收入7245元，增长11.1%。落实“零就业家庭援助计划”，启动小额贷款担保工作，大力推进农村富余劳动力转移和新成长劳动力就业与创业，农村富余劳动力就业20512人。城镇登记失业率控制在2.5%以内。调整社保缴费基数和待遇，全市参加社保五大险种总达311.8万人次，社保基金征缴率达99.7%，养老金社会化发放率100%。积极做好农村基本养老保险实施前的准备工作,试点工作取得良好效果。设立全市扶困助学基金，已筹集资金705.2万元。推进“安全五项工程”。城乡水利防灾减灾工程投入3.5亿元，防灾减灾能力进一步增强。完成四类桥（涵）改造102座。完成4.5万平方米城区C级公房危房改造和住户安置工作，新建解困小区3.8万平方米。对全市农村188户低保家庭危房进行改造。加快解决教师、公安干警和医生住房问题。抓好疾病防控工程建设。加快绿色食品基地建设，加强农产品安全监测。大力推进水环境综合整治，启动生态市创建工作，编制《中山市生态市建设规划》，加强城市环境综合整治，加快“两控区”（烟尘控制区和噪声达标区）创建步伐，积极实施治污保洁工程，开展清理整顿沙石泥场、实心粘土砖厂和清查放射源及固体废弃物专项行动，加强医疗废弃物管理，推进清洁生产企业试点。我市成为“国家环保模范城市”免检城市。

加强政府自身建设。贯彻实施《行政许可法》，清理行政许可实施机构217个，保留109个，变更实施主体38个，清理市政府规范性文件255件。大力推进依法治市，坚持依法行政，全面推行行政执法责任制和过错责任追究制度。实施政府重大事项听证制度，重大事项向市委、市人大报告制度，与市人大常委会、政协领导分别就农产品安全和水资源保护问题进行了座谈。切实抓好政府行政效率提速，加快电子政务建设。推进以政府“网上审批”系统建设为重点的新一轮审批制度改革，共有32个部门136个审批事项上网运行，网上受理办结1.6万多宗，政务信息化水平进一步提高。推进政府工作公开化、透明化。加强公务员队伍建设，扎实推进廉政建设和反腐败斗争，继续开展机关作风评议和争当“人民满意公务员”活动。坚持政府新闻发布制度，加大政府督查及投诉处理力度，行政效率得到提升。

民政、监察、外事、侨务、台务、审计、统计、老干、信访、广播电视、气象、国防建设、档案、地方志、妇女儿童、残疾人、民族宗教、新闻出版、人防、老区建设等各项工作取得了新的成绩。

各位代表，过去一年的成绩来之不易，这是省委、省政府和市委正确领导的结果，是全市人民共同奋斗的结果，也是各位人大代表、政协委员监督、支持的结果。我谨代表市人民政府向全市广大工人、农民、知识分子、干部职工、驻我市人民解放军指战员、武警官兵、人民警察和各界人士表示崇高的敬意，向长期关心支持中山建设的港澳台同胞、海外侨胞和国内外友人表示衷心的感谢！

我们要清醒地认识到，我市经济社会发展还存在一些深层次矛盾和问题。主要是：粗放型经济增长方式没有实现根本转变，产业优势不明显，结构有待优化，产业之间、区域之间的发展不够协调；中心城区的集聚辐射功能不强；公共管理、公共服务与经济发展的水平不相适应；维护社会稳定方面还有大量工作要做；社会信用体系亟待进一步完善等。这些问题都需要我们在今后的工作中进一步解决。

2005年政府工作的总体安排

2005年是为全面加快率先基本实现现代化奠定坚实基础的重要一年，做好今年的工作意义重大。当前，我市经济社会发展总体形势向好，经济运行质量正在逐步提高，为我市加快发展带来了前所未有的机遇；但是，日趋激烈的区域竞争，以及在发展中逐步解决深层次矛盾和问题的压力，也给我市带来前所未有的挑战。必须以时不我待、只争朝夕的精神，用发展的眼光、创新的办法和统筹的思路，按照科学发展观和五个统筹的总体要求，在把握大局中谋划未来，在协调发展中彰显特色，力争发展得更快更好，努力开创经济和社会发展新局面。

2005年政府工作的总体要求是：以邓小平理论和“三个代表”重要思想为指导，全面贯彻党的十六大和十六届三中、四中全会、中央经济工作会议、胡锦涛总书记视察广东重要讲话、省委九届六次全会和市委十一届二次全会精神，牢固树立和落实科学发展观，坚持把发展作为第一要务，按照五个统筹的要求，加快经济结构调整，转变经济增长方式，努力实现经济增长质量、效益同步提高；大力推进社会主义精神文明建设，全面加快社会事业发展，提高全社会的文明程度和市民的道德文化素质；加强社会主义民主法制建设，坚持依法行政、民主行政和科学行政，全面提高政府的公共服务、公共管理水平；完善城市功能，增强城市综合竞争力；坚持以人为本，注重社会公平，建设既适宜居住又适宜创业的美好家园，努力构建和谐中山。

2005年全市经济和社会发展的预期目标为：全市生产总值增长12%；工业总产值增长15%；工业增加值增长15%；全社会固定资产投资增长12%；社会消费品零售总额增长12%；外贸出口总值增长8%；城镇居民年人均可支配收入增长8%，农民人均年纯收入增长8%；城镇登记失业率控制在3%以内；人口自然增长率控制在6.5‰以下。

为实现上述目标，必须着力抓好以下工作：

一、加快产业结构调整，全面提升产业竞争力

转变经济增长方式，提高经济增长质量。抓住国际资本与产业加速转移，我国经济进入新一轮增长周期、区域合作趋势更加明显的机遇，加快经济结构调整，切实转变经济增长方式，努力提高经济增长质量。坚持走新型工业化发展道路，按照“高、重、集、内”的思路（加快发展高新技术产业，积极引进资金、技术密集型重型化工业和装备制造业，促进产业集聚，大力发展内源型经济），按照新一轮产业发展目录，加快促进经济增长方式由粗放型向集约型、循环型转变，努力走出一条科技含量高、经济效益好、资源消耗低、环境污染少、人力资源优势得到充分发挥的产业发展之路。

促进产业集聚，提升发展水平。坚定不移地推进“工业强市”战略，促进产业配套对接，优化产业发展环境，提升在区域分工体系中的地位，促进从加工基地向制造服务基地转变。通过产业聚集、优化和提升，促进产业结构不断向合理化、高级化、重型化发展，加快建设产业强市。逐步形成以四大组团为增长极，18个国家级专业产业基地和各类园区为增长点，东部沿海为高新技术产业带的产业特色。进一步增强各类工业园区集聚资本、吸纳人才、吸收资金的能力，以园区为依托，实现“园镇互动”，集约发展。引导园区合理安排开发时序，鼓励园区错位发展，加大整合、提升力度，做到“统一规划、滚动发展、集约建设”。严把企业入园关，提高工业用地投资密度、产出效益和环保效益的准入门槛。大力推进工业示范园区建设工作。

加快发展装备制造业，完善产业体系。积极引进和发展资金、技术密集型的重型化、高加工工业和装备制造业。不断完善扶持装备制造业发展的政策措施，把装备制造业作为工业发展的基础性和战略性产业，安排2000万元作为专项资金，“科技三项”经费和技改资金的50%以上用于扶持装备制造业，重点发展汽车主要零部件、输配电及控制设备制造、专用设备制造、电子及通讯设备制造等，逐步形成产业群。强化产业带动效应，形成更具竞争力的产业发展体系。促进各类要素资源向优势产业集聚，加快发展电子信息、健康医药、包装印刷等优势产业，培育壮大新材料、新型环保、光机电一体化等高成长性产业。优先发展高新技术产业，积极应用高新技术、先进适用技术和信息技术改造传统产业，促进产品升级换代。

大力发展中小企业，增强特色产业集群优势。安排1000万元用于扶持中小型企业特别是民营企业的发展，重点突破创新瓶颈，加快企业机制创新、技术创新。鼓励中小企业走专、精、特、新的发展道路，提高为大型企业配套生产的能力。结合组团发展优化产业布局，加快建立区域产业协作体系，按照“规模优势明显，产业特色突出，创新创业活跃”的要求，做大做强专业产业基地，催生新的专业基地，加快生产集约化和经营集团化步伐，促进特色产业从生产环节向研发设计和营销品牌等环节延伸，逐步实现从专业化产业集群到区域创新体系的转变。各专业镇和专业生产基地要创新发展模式，从专业化分工、集群化布局、市场化联动、社会化协作方面构建新的发展平台，提升发展水平和发展后劲。

实施名牌战略，增强企业核心竞争力。安排专项资金用于名牌、名标奖励，争创一批新的名牌、名标，加大对名优品牌的保护力度，引导生产要素向名牌产品和优势企业集中，以名牌促进市场开拓和产业发展。注重产品质量水平的不断提升，重点扶持发展一批以名牌产品为核心，主导产品优势突出的骨干企业。不断巩固和发展区域品牌，打造“中山制造”的整体品牌形象。

发展循环经济，构建节约型产业结构。坚持理性发展的思路，把节约能源、资源作为优化产业结构的重要目标。大力研究和发展循环经济，推广清洁生产，加快节能新技术、新工艺、新设备的推广应用，探索研究再生资源回收利用产业的发展。优化固定资产投资方向和产业投向结构，努力建设低投入高产出、低消耗少排放、能循环可持续的新型经济增长体系、节约型产业结构和节约型社会。

二、进一步完善农业、农村发展支持和保障体系，加快城乡一体化进程

坚持农业基础地位不动摇，增强农业发展能力。全面贯彻中央一号文件精神，进一步落实“多予、少取、放活”的方针，加大工业对农业的反哺力度，逐步建立和完善以工促农、以城带乡的发展新机制，合理调整财政支出结构，增加农业投入。继续推进农村“五项工程”和农田基本建设，努力改善农村生产生活条件。进一步恢复和提高粮食综合生产能力，确保粮食安全。推动渔业和林业加快发展。

深化农业结构调整，提高产业化水平。促进农业增长方式的根本转变和农业节本增效，走大规模、深加工、大市场的路子，充分挖掘农业内部增收潜力。加快结构的战略性调整，按照优质、高产、高效、生态、安全的要求，大力发展特色农业、品牌农业和绿色农业。增强农业科技创新能力，积极推进区域化、专业化、规模化生产和集约化经营。落实好省、市扶持农业龙头企业发展政策，扶持一批农业龙头企业，通过龙头带动和利益联动环节，提高农业产业化水平。鼓励农民成立各类生产、经营合作组织，提高农民生产组织化程度和农业社会化服务水平，逐步实现基地化、订单化、标准化生产。推广农业科技，办好水利园区和农业科技示范园区。加快农村市场体系建设，大力发展现代流通方式，开辟农产品“绿色通道”，控制农业生产成本上升。健全农产品检验检测网络，提高农产品的质量，确保食品安全。要切实加强动植物疫病防控工作。

深化农村各项改革。推进农村“固本强基”工程。加强村务公开、民主管理。推进镇村公共财政制度，降低镇村行政成本，化解镇村债务。加强农村集体土地和征地款管理，解决征地农民生活保障问题。加大力度推进农村集体经济股份合作制。总结推广小榄镇村改居和古镇村一级核算的经验，鼓励有条件的镇区推进村改居工作，形成农业和农村经济良性发展机制。巩固农村税费改革成果，全面免征农业税，确保农民持续增收。

加快城镇化步伐。统筹城乡劳动力市场、义务教育和公共卫生事业发展。加快农村劳动力向二、三产业转移，安排800万元作为再就业培训专项经费。积极推进户籍制度改革，鼓励农民进城就业、定居，鼓励农民异地开发、转移发展。加强镇村规划建设管理，引导有序发展。大力发展城镇公用事业，按照政府引导、规划先行、建设有序、市场运作、产业支撑的思路，以产业集聚为基础，完善城镇功能，实现人口向城镇集中、住宅向社区集中。加快中心镇、中心村建设，形成市、镇、村三级联动，加快城中村改造，引导农民向城市居民转变。大力推进农民居住小区规划建设。

三、实施重点工程带动，提升城市综合服务功能

积极参与珠三角城市群的分工和协调发展，拓展城市发展空间。完成全市总体规划修编和重点专项规划编制，进一步拉开城市框架，优化基础设施建设和城市功能的空间布局，构建新一轮发展平台。创新发展模式，整合组团资源，促进镇区间的平衡发展和城市基础设施的有效共享，加快推进东部沿海地区开发和西北组团建设。努力实现在建城、修路、护山、治水、增绿、控污等城市建设的各个方面取得更大成效。

全面推进重点工程建设。适度超前发展基础设施，筹集资金加快建设一批关系全市发展全局的功能性、枢纽性项目。实行重点工程预备项目库制度，成立重点工程领导机构，统筹协调项目建设的论证、立项和启动工作。在交通方面，配合省做好轻轨、铁路和“两纵两横一环”高速公路网的规划建设。加快市域路网“内通外拓”，加强与周边城市道路网络对接，推进“五纵五横”规划建设，加快建设福源路、南外环路、北外环路、古神公路、城桂路二期及岐江公路改造。强化中心城区的辐射带动和综合服务功能，优化路网结构；完善连接外环的配套路网建设，整治城区出入口；改造博爱路、起湾道等城区交通主干线，分期整治莲员路、清溪路；按照公交优先的思路，建设一批公交枢纽站和停车场，构建安全、便捷、高效的公交网络。在能源方面，扩建500千伏香山站，新建和扩建220千伏迪光站和11座110千伏变电站。在环境建设方面，加快中嘉污水处理厂二期、珍家山污水处理厂首期、中心组团垃圾综合处理基地焚烧厂建设，建设岐江广场、马山公园、古香林郊野公园。在城市防灾减灾工程建设方面，加快大涌口水闸、田基沙水闸等建设工程。

严格土地管理，提高土地利用率。土地是不可再生资源，必须牢固树立“珍惜和利用好每一寸土地”的思想，按照“建立最严格的土地管理制度”的要求，切实转变土地开发、经营方式，提高土地投入和产出效益，实行土地一级市场垄断经营。加大国土资源的执法和监督力度，细化“批项目、控土地”制度。加大力度整合和盘活闲置土地，强化土地资源储备，严格耕地保护。

提升城市公共管理和服务水平。强化城市经营理念，坚持“以地建城”，推动城市资产滚动发展。探索项目融资、银行融资、股票市场融资、公用设施冠名权、特许经营等产权置换方式筹集城市建设资金。强化基础管理工作，健全城市公共设施营运、维护和管理机制，发挥公共设施最大效益。建立相互衔接、合理分工和规范高效的城市建设和管理综合执法体制，提高城市综合管理水平。

四、内源型与外源型经济并重，增强经济自主增长能力

扩大对外开放，提升外源型经济水平。根据国际资本流动的趋势和特点，实现从招商引资到招商选资的转变；从优惠政策招商向投资环境、产业配套、市场优势和制度优势招商并重的转变；从引进资金到引进资金、人才、技术并重的转变。不断创新招商形式，构建政府引导的社会化、专业化、市场化招商网络，积极探索有效的海外招商模式，大力推动企业招商。重点引进欧美和日韩大型企业，积极争取生产原材料、装备工业等上游产业的转移性投资。鼓励外商投资高新技术产业、先进制造业、现代服务业、现代农业和环保产业。积极引进跨国公司特别是世界500强企业，鼓励跨国公司到中山设立研发中心、地区总部和先进生产制造基地。鼓励现有外资企业增资扩产。做好2005年招商经贸洽谈会各项工作，积极组织参加广交会以及各类大型会展。

转变外贸增长方式，推动市场和贸易方式多元化。推进科技兴贸、名牌带动战略，促进出口商品结构和加工贸易转型升级，扩大高新技术产品、机电产品和农产品的出口。进一步扩大和优化外贸经营主体，鼓励有条件的企业“走出去”与国内外企业优势互补、联合发展。推动对外经贸合作从引进制造业为主向引进制造业和服务业并重发展。推广国际质量标准体系，建立高效的反倾销及各种贸易壁垒的预警机制，有效规避风险。

营造良好的发展环境，加快内源型经济发展。积极吸引国内大企业来中山投资。落实好省、市在鼓励、扶持民营企业创业和发展方面的政策和措施，重点扶持一批科技型、外向型、农产品加工型的民营企业做大做强；鼓励民营企业建立和完善现代企业制度。加大力度推进优质民营企业上市。完善民营企业服务体系，大力营造创业氛围，培育和造就一大批优秀企业家。

参与区域合作，超常规发展第三产业。发挥“东西结点，南北逢源”的区位优势，按照“共享、共赢和错位互补”的原则，积极引进港澳高端服务业，重点发展为生产服务的现代物流业，壮大连锁经营和中高级批发市场，申报保税物流园区。推进区域性旅游协作，整合旅游资源，促进旅游业的深度开发。引导房地产业的有序健康发展，规范物业管理。大力发展中介服务业。提高城乡居民消费能力，加快消费结构升级，培育信息、汽车、住房、文化、健康等新型消费热点，增加服务性消费。探索建立和完善个人征信系统，扩大信贷消费规模。继续推进金融、证券、保险业健康发展。办好专业会展，大力开拓国内市场。

继续大力整顿市场经济秩序。开展各类专项整治，严厉打击走私贩私、制假售假、偷逃骗税、非法传销、商业欺诈等违法犯罪行为，营造良好的市场环境。加快培育行业协会、商会和市场中介组织，推动社会信用中介机构的发展。

五、坚持深化改革，加快体制创新，增强机制活力

完善公有资产管理，提高监管水平。着眼于增强活力和竞争力，继续深化公有企业改革，探索建立职业经理人制度。强化市属及市控股公司的经营管理，建立以利润为目标的经营管理责任制，确保国有资产安全、保值和增值。巩固公有企业改革成果，继续做好“一改二清”（国有企业转制改革、清算销户、清减债务）工作。健全国有资产监督管理体系，完善公有企业经营业绩考核和国有资产收益收缴办法，提高监管水平。

创新财税管理机制。切实加强税收征管，依法治税。积极组织非税收入。各级财政要按照公共财政的要求，继续树立“过紧日子”的思想，做到生财有道、聚财有方、用财有规，抓好增收节支，狠抓收入管理，提高支出效益，确保财政稳健运行。要强化预算约束，提高各级理财水平，严格控制一般性支出。逐步实现专项支出项目的动态管理。完成市级预算单位全面纳入国库管理工作。强化财政支出绩效评价工作，逐步引入项目绩效预算制度。建立转移支付和分类管理制度，增强镇区财政抗风险能力，年度安排的转移支付资金不少于1亿元。进一步加强政府采购工作。

深化投融资体制改革。大力发展资本市场，完善市场体系。逐步开放基础设施项目投资、建设、管理和运营市场，鼓励民资、外资参与基础设施建设。通过市场选择投资主体。实行政府公益性项目代建制。鼓励多种投资主体参与社会公用事业领域的投资。公共资源经营统一纳入政府资源管理。加快中小金融机构退出市场工作。积极利用资本市场，帮助各类企业增强进入资本市场直接融资的能力。

积极推进科技、教育、文化、体育、卫生等各项改革。

六、统筹经济社会发展，加快建设文化强市步伐

增强科技创新力。把提高自主创新能力作为推动结构升级的中心环节，坚持引进和消化、吸收、创新相结合，注重对引进技术的二次开发，大力增强科技创新能力和核心竞争力。安排“科技三项”经费1830万元，安排技改资金3100万元。要提高科技投入使用绩效，发挥财政资金的放大效应，引导更多社会资金参与科技项目建设，努力提高科技综合实力、创新能力和辐射力。扶持各级各类工程中心、技术中心、生产力促进中心等研发机构的发展，推动科技专业镇建设。完善科技中介服务机构，支持和鼓励风险投资，探索和完善风险投资的市场化运作机制。加快构建产权交易平台，推进产学研一体化进程，鼓励企业在装备制造、信息、环保、生物和新材料等领域，引进和开发一批联动效益大、支撑产业升级的科技项目。办好“中国技术市场科技成果产业化（中山）示范基地”，做好中科院生物医药科研成果向健康科技产业基地转化工作。加强知识产权保护，奖励专利开发。继续做好科普工作。

实施人才强市战略。全面加强人才建设，发展专业化、信息化、产业化的人才市场服务体系，促进人才合理流动。以产业发展为依托，通过系统开发、柔性流动建设人才集聚工程，多形式、多渠道引进和培养高层次、复合型、高技能人才和紧缺专业人才，形成吸引人才、留住人才、发挥人才作用的良好氛围。创新人才培养模式，逐步建立国民教育和终身教育互动的人才培养体系。完善人才评价机制和人才奖励制度。

加快教育事业发展，实现教育强市目标。加快创建教育强镇步伐，确保教育强市目标如期实现。创新办学体制，大力发展民办教育，探索股份制办学、组建教育集团等办学方式。推进“三名”工程，完成校长任期目标管理和绩效评价制度建设。促进各级各类教育协调发展，全面推进振兴初中行动计划，建设中山职业技术学院和高级技工学校，大力支持发展高等教育事业。提高教育信息化水平。加大教育投入，安排贴息资金，协助镇区完成中小学布局调整任务，实现基础教育均衡发展。

加强公共卫生建设，不断提高城乡居民健康水平。加强传染病特别是重大传染病的防控和卫生监督工作，完善公共卫生网络体系，增强公共卫生事件应急处理能力。提高医疗卫生工作质量。优化卫生资源配置，推进镇、村医疗卫生一体化管理，在镇区建立防保所。积极拓展社区卫生服务，有序放开医疗市场。加强医疗收费监察和管理，深入开展健康教育和爱国卫生运动，引导广大群众形成健康文明的生活方式。

大力发展文化事业和文化产业。坚持“以经济强筋骨，以文化论输赢”的发展理念，深入挖掘历史文化资源，彰显城市的文化内涵。继续做好文物保护和修缮工作，完成文化艺术中心建设，加强农村文化设施建设。丰富和发展广场文化、社区文化等多种形式的群众性文化艺术活动。发展文化经济，壮大文化产业。大力发展体育事业，推动全民健身运动深入开展，不断提高竞技体育水平。

发展各项社会事业。深化人口计生综合改革，合理控制人口总量与布局，形成适应产业发展需要的人口结构。加快发展社会福利和残疾人事业，支持慈善事业发展，做好妇女、儿童、老龄工作。加强国防教育和民兵预备役建设，做好“双拥”和优抚安置工作。

七、努力创建文明法治谅解宽容社会环境，构建和谐中山

以争创全国文明城市为契机，全面提升市民文明素质。大力开展群众性主题教育实践活动，加强社会公德、职业道德和家庭美德教育，积极开展“爱国、守法、诚信、知礼”现代公民教育活动，弘扬“博爱、创新、包容、和谐”的新时期中山人精神。引导群众学习文化、崇尚科学、破除迷信、移风易俗。深入开展群众性精神文明创建活动，以文明社区、文明村、文明镇等各项创建活动为基础，进一步增强各项创建活动的覆盖面和影响力，弘扬注重竞争与效率、促进公平与正义、提倡谅解与宽容的社会风尚，提升市民文明道德水平，为建设文明城市奠定坚实基础。大力推进“诚信中山”建设，继续整合社会信用资源，完善社会信用体系，充分发挥中山信用网对工商企业信用信息的披露作用，逐步建立全社会的信用监管制度。

优化生产生活环境，建设“绿色中山”。努力培育环境友好、资源高效、系统和谐的生态文明形态。设立五桂山生态保护区，全面开展创建全国生态市建设。推进水环境保护工作，抓好内河整治，改善两岸景观，打造“健康河流”。加强全市“供水一盘棋”规划的实施和分质供水规划研究，加强旧城区和“城中村”管网改造，确保饮用水安全。加大“三废”治理和治污力度，确保空气质量稳定，加快各组团垃圾处理基地和医疗废弃物处理中心建设，大力推进镇区污水处理设施建设。全面启动新一轮“绿化中山大地”工程，积极开展“四旁”绿化、道路绿化改造、林相改造、郊野森林公园建设和五桂山森林资源保护工作。做好创建“绿色交通示范城市”的各项工作。

扎实推动各项“民心工程”。继续抓好省政府“十项民心工程”的实施。积极扩大城乡就业，建设全市劳动力中心市场，全面完成三年内转移5万农村富余劳动力的任务，确保新成长劳动力100%接受培训，100%实现就业。完善下岗失业人员自主创业培训政策，提高小额贷款标准，实施“零就业家庭”帮扶计划。投入1.1亿元大力推行社会保险事业，努力构建完善的社会保障体系。做好巩固社保扩面工作，实现社会保险参保320万人次以上。大力推进农村基本养老保险，年内实现20万以上农民参保目标。调整和提高社会保险缴费和待遇，提高保障水平。巩固农村合作医疗成果，探索向农村医疗保险的过渡与衔接。努力维护社会公平，健全社会化救助体系。严格落实“低保”政策，安排低保资金1300万元。加强低收入困难家庭的社会救济工作，帮助困难群众解决生活、就业、医疗、住房、子女入学等方面的实际困难。市财政安排专项资金启动全市扶困助学基金工程。健全法律援助制度，做好劳资纠纷预防和处置工作，切实解决建设领域拖欠工程款问题，维护劳动者的合法权益。继续推进“五项安全工程”，积极创新投入机制，投资4.6亿元开展83宗城乡水利防灾减灾工程建设项目；推进食品质量安全四大体系建设，推行食品市场准入管理；加快危桥（涵）改造进度，提高管养水平；推进全民安康工程，加快第二人民医院和市疾控中心建设进度。努力使全市人民喝上干净的水、呼吸清新的空气、吃上放心的食物、住上舒适的房子，在良好的环境中工作和生活。

加强利益协调，促进社会公平与社会稳定。深入开展“四五”普法教育，积极做好人民调解工作。创新信访工作机制，最大限度地预防和化解社会矛盾，积极预防和妥善处置群体性事件。加快建立联动机制，健全防范突发事件、重大自然灾害及社会风险的预警、防范和应对机制。加强社会治安综合治理，加强外来流动人口及出租屋管理，提高控制能力，严厉打击各种刑事犯罪，广泛开展创建“和谐平安社区”活动。强化全社会的安全生产意识，大力推进安全生产监管体制、执法队伍和法制建设，构建安全监管网络，进一步落实安全生产责任制。严格安全生产许可制度，强化日常监管，确保安全生产形势稳定。

抓紧做好“十一五”规划的制定工作。坚持以科学发展观为指导，用改革的精神、创新的思路、科学的方法进行规划编制工作，把以人为本、全面协调可持续的发展理念贯穿规划始终，突出重点任务、重点领域、重点区域和重大工程，努力使“十一五”规划成为体现“五个统筹”、促进经济社会全面协调可持续发展的规划。

建设廉洁勤政高效务实政府

坚持依法行政、民主行政和科学行政，提高行政水平。全面落实《行政许可法》，规范许可项目，明晰实施主体，严格依照法定权限和法定程序行使权力，健全行政决策监督和责任追究制度。认真贯彻《全面推进依法行政实施纲要》，加大行政执法检查力度，严格执行行政执法过错追究责任制度。做好行政复议和应诉工作，妥善解决行政争议。主动接受市人大及其常委会的法律监督，政协、各民主党派、工商联、无党派人士和人民团体的民主监督，接受公众和舆论监督，认真听取意见，提高人大代表建议和政协委员提案的办理质量。积极推进要素市场、建筑、土地市场的规范运作。推进政务公开制度化、规范化建设，打造政务“阳光工程”。通过完善政务公开、厂务公开、重大决策公开听证、专家咨询等，进一步保障人民群众的知情权、参与权、选择权和监督权。确保决策的民主化和科学化，深入推进“诚信政府”建设，提高政府公信力，建设人民满意政府。

提升行政管理效能，优化发展环境。更加重视区域经济发展从政策竞争向制度竞争转变的大趋势，进一步提高政府的社会管理和公共服务水平，推进政府行政向公共行政转变，真正把政府职能转变到经济调节、市场监管、社会管理和公共服务上来，既要强化管理职能，更要提供优质服务，着力推动法制、政策、人才、信用、服务等环境的建设。坚持以人为本，推进公共行政体制的创新，不断优化工作流程，努力形成行为规范、运转协调、公正透明、廉洁高效的运行机制，切实防止在职能履行中越位、错位和缺位。不断提高职能履行水平，形成顺畅的部门沟通和协调机制，实现政府的高效管理。强化服务意识，积极创树机关服务品牌，深入开展行政审批制度改革，缩短审批时限，降低行政成本。以中山市行政服务在线建设为载体，继续加大力度整合行政资源，实现更多行政审批事项“网上办理”，大力推进政府行政与社会管理的信息化应用。

从严治政，建设高素质的公务员队伍。加强公务员宗旨、信念教育，树立廉政、高效的行政新风，坚持“两个务必”和“权为民所用、情为民所系、利为民所谋”的行政理念。继续开展机关作风评议和行风评议活动，坚决纠正侵害群众利益的不正之风，从源头上防治腐败。建立和健全与社会主义市场经济体制相适应的教育制度，监管并重的惩治和预防腐败体系，坚决查处各种违法违纪的大案要案。从严治政，力戒骄惰散漫之气。建立健全行政绩效评估机制，提高政府部门及其工作人员的服务效率和质量，加大行政效能监察和对行政不作为、乱作为的责任追究力度。努力培养学习型、创新型、服务型和干净干事的公务员队伍。

各位代表，今年是全面实现“十五”计划，衔接“十一五”发展规划的重要一年，是推进全面建设小康社会和经济社会协调发展示范市的重要一年。立足当前，我们信心百倍；展望未来，我们深感责任重大。让我们高举邓小平理论和“三个代表”重要思想伟大旗帜，牢固树立和落实科学发展观，在省委、省政府和市委的领导下，开拓创新，与时俱进，励精图治，为建设既适宜居住又适宜创业的美好家园，构建和谐中山，实现富裕安康而努力奋斗！
